# Right ventricular energy metabolism in a porcine model of acute right ventricular pressure overload after weaning from cardiopulmonary bypass

**DOI:** 10.14814/phy2.15421

**Published:** 2022-11-16

**Authors:** Masaki Kajimoto, Muhammad Nuri, Justin R. Sleasman, Kevin A. Charette, Hidemi Kajimoto, Michael A. Portman

**Affiliations:** ^1^ Center for Integrative Brain Research Seattle Children's Research Institute Seattle Washington USA; ^2^ Division of Cardiothoracic Surgery at Children's Hospital of Philadelphia Philadelphia Pennsylvania USA; ^3^ Division of Pediatric Cardiac Surgery Lucile Packard Children's Hospital Palo Alto California USA; ^4^ Division of Pediatric Cardiac Surgery Seattle Children's Hospital Seattle Washington USA; ^5^ Division of Cardiology, Department of Pediatrics University of Washington Seattle Washington USA

**Keywords:** cardiopulmonary bypass, congenital heart disease, myocardial metabolism, right ventricular pressure overload

## Abstract

Acute right ventricular pressure overload (RVPO) occurs following congenital heart surgery and often results in low cardiac output syndrome. We tested the hypothesis that the RV exhibits limited ability to modify substrate utilization in response to increasing energy requirements during acute RVPO after cardiopulmonary bypass (CPB). We assessed the RV fractional contributions (Fc) of substrates to the citric acid cycle in juvenile pigs exposed to acute RVPO by pulmonary artery banding (PAB) and CPB. Sixteen Yorkshire male pigs (median 38 days old, 12.2 kg of body weight) were randomized to SHAM (Ctrl, *n* = 5), 2‐h CPB (CPB, *n* = 5) or CPB with PAB (PAB‐CPB, *n* = 6). Carbon‐13 (^13^C)‐labeled lactate, medium‐chain, and mixed long‐chain fatty acids (MCFA and LCFAs) were infused as metabolic tracers for energy substrates. After weaning from CPB, RV systolic pressure (RVSP) doubled baseline in PAB‐CPB while piglets in CPB group maintained normal RVSP. Fc‐LCFAs decreased significantly in order PAB‐CPB > CPB > Ctrl groups by ^13^C‐NMR. Fc‐lactate and Fc‐MCFA were similar among the three groups. Intragroup analysis for PAB‐CPB showed that the limited Fc‐LCFAs appeared prominently in piglets exposed to high RVSP‐to‐left ventricular systolic pressure ratio and high RV rate‐pressure product, an indicator of myocardial oxygen demand. Acute RVPO after CPB strongly inhibits LCFA oxidation without compensation by lactate oxidation, resulting in energy deficiency as determined by lower (phosphocreatine)/(adenosine triphosphate) in PAB‐CPB. Adequate energy supply but also metabolic interventions may be required to circumvent these RV energy metabolic abnormalities during RVPO after CPB.

## Graphical abstract

Acute right ventricular pressure overload (RVPO) after cardiac surgery using cardiopulmonary bypass (CPB) is associated with inceased risk of RV failure. We found that (1) acute RVPO after CPB strongly inhibited FA, specifically long‐chain fatty acid (LCFA), oxidation in RV, and the FA metabolic abnormality correlated to RV‐to‐LV systolic pressure ratio inversely, and (2) lactate oxidation did not accelerate in response to the decreases in LCFA oxidation. These metabolic abnormalities can result in energy deficiency in RV under RVPO after CPB.

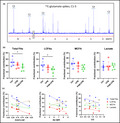

## 1 INTRODUCTION

Acute right ventricular (RV) failure frequently occurs in infants and children immediately following surgical procedures directed at repair or palliation of congenital heart defects (Bolger et al., 2002; Graham et al., 2000; Norozi et al., 2006; Reddy & Bernstein, 2015). The RV is often exposed to new or persistent pressure overload due to obstruction to pulmonary blood flow, pulmonary hypertension, or maintenance of the RV as the systemic ventricle (Celermajer et al., 1993; Levy et al., 2021; Schulze‐Neick et al., 1999). Palliation of defects involves cardiopulmonary bypass (CPB) and often aortic cross‐clamping and reperfusion, which superimpose additional stress on both ventricles. Additionally, the usage of CPB induces an intense inflammatory response (Tu et al., 2021) and is associated with a variable degree of vascular reactivity leading to acute pulmonary hypertension and RV pressure overload (RVPO) (Celermajer et al., 1993). These processes occur during CPB onset and continue through weaning and the acute phase of post‐CPB. The mechanisms responsible for contractile dysfunction of the immature RVPO still need elucidation in order to develop targeted therapies (Haller et al., 2020). Data from recent studies imply that RV inability to modify substrate flux contributes to energy imbalance and results in contractile failure after acute RVPO. Some data suggest that chronic RVPO promotes metabolic changes which lead to maladaptive hypertrophy in the RV (Koop et al., 2019; Piao et al., 2010; Prisco et al., 2022; Sheikh et al., 2009). Furthermore, reversal of these metabolic perturbations, which include alterations in substrate utilization, prevents or limits functional decompensation. Thus, some investigators have proposed using metabolic strategies to treat the RV failing due to pressure overload (Kajimoto et al., 2018, 2019; Piao et al., 2010; Sun et al., 2016). Though this treatment paradigm shows some potential, the limited existing data still need substantial validation and vetting in clinically applicable experimental models emulating conditions unique to the newborn infant undergoing congenital heart surgery. Prior studies did not evaluate additional stressors such CPB superimposed on pressure overload (Kajimoto et al., 2018, 2019). In this study, we tested the hypothesis that the RV exhibits limited ability to modify substrate utilization in response to increasing energy requirements during acute RVPO after CPB in juvenile pigs.

## 2 MATERIALS AND METHODS

### 2.1 Animals and experimental design

**FIGURE 1:**
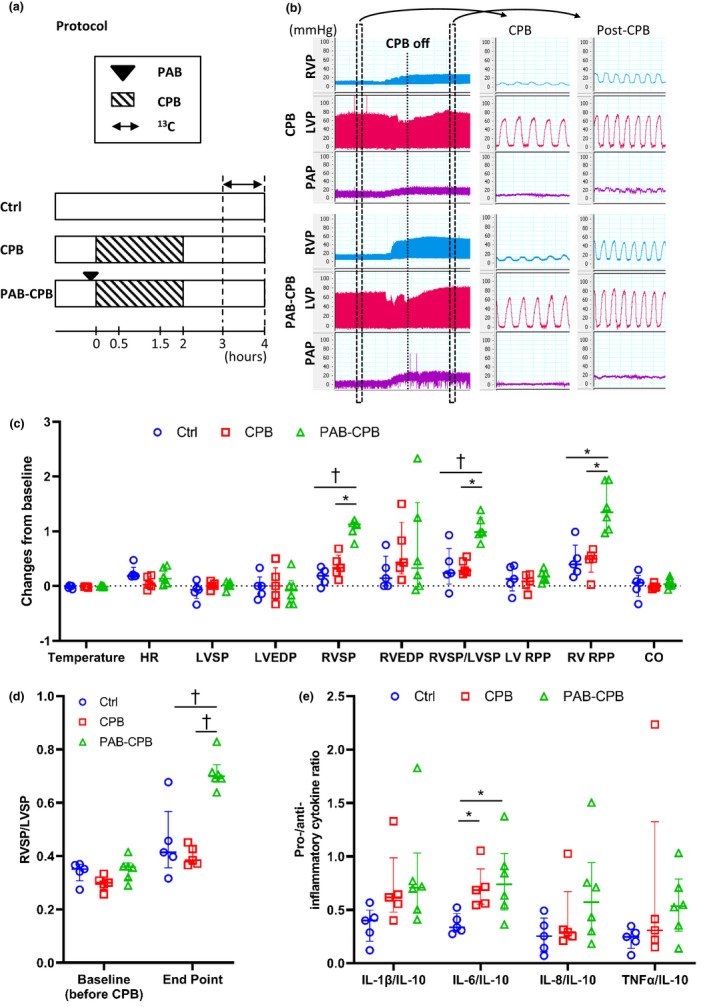
Diagram of the experimental protocol and Cardiac parameters. (a) The durations of CPB and Carbon‐13 (^13^C)‐labeled substrate infusion were 120 and 60 min, respectively. Heart tissue was collected at the end of the infusion. (b–d) Cardiac parameter is RVSP, RVSP/LVSP and RV RPP markedly increased in PAB‐CPB group. (e) The usage of CPB induced high IL‐6/IL‐10. The proinflammatory cytokines/IL‐10 ratio were similar in CPB and PAB‐CPB groups. Values are median (interquartile range); *n* = 5–6 per group. *p < 0.05; ^†^ *p* < 0.01. CO, cardiac output; CPB, cardiopulmonary bypass; EDP, end diastolic pressure; HR, heart rate; IL, interleukin; LVP, left ventricular pressure; PAB, pulmonary artery banding; PAP, pulmonary arterial pressure; RPP, rate‐pressure product; RVP, right ventricular pressure; RVSP/LVSP, RV‐to‐LV systolic pressure ratio; TNF, tumor necrosis factor.

Experiments were carried out in 1 month‐old male mixed breed Yorkshire piglets with median weight of 12.2 (2.1) kg (*n* = 16). Our study models were designed for acute RVPO in post‐CPB as shown in Figure 1a. Acute RVPO in post‐CPB were intentionally induced by pulmonary artery banding (PAB). Piglets were randomized to 3 groups; Ctrl group (without CPB, *n* = 5), CPB group (2‐h CPB, *n* = 5), and PAB‐CPB group (PAB before 2‐h CPB, *n* = 6). Piglets in PAB‐CPB group were exposed to acute RVPO immediately after weaning from CPB. Once stable vitals of pigs were established after the completion of CPB support (at around 30 min after weaning off CPB), Carbon‐13 (^13^C)‐labeled substrates were infused into the right coronary artery. Heart tissue was harvested immediately upon completion of the ^13^C‐labeled infusion for 60 min, and rapidly stored under liquid nitrogen for later extraction. All experimental procedures in this study were approved by Seattle Children's Institutional Animal Use and Care Committee.

### 2.2 Perioperative and CPB management

Perioperative management has been described previously in detail (Kajimoto et al., 2013, 2016, 2017). After appropriate sedation and anesthesia, pigs were intubated through a surgical tracheostomy and were mechanically ventilated with oxygen (40%–50%) and isoflurane (1%–2%) mixture. Pigs have a right cranial lobe (tracheal) bronchus, which arises from the right wall of the trachea. Instead of endotracheal intubation, we performed a surgical tracheostomy that can definitely prevent obstruction of the bronchus. In this study, monitors were placed for electrocardiogram, oxygen saturation, temperatures of esophageal and rectal, the flow of aorta and pressures of the femoral artery, central vein, PA, RV and left ventricle (LV). A PowerLab 16/30 recorder (AD Instruments) was used to continuously record these data throughout all protocols. Rate‐pressure product (RPP) was calculated as the product of heart rate (HR) and systolic blood pressure (SBP), HR × SBP/1000. Cardiac output (L/min) was calculated from aortic flow data that was measured by a flow probe (Transonic Systems Inc) around the ascending aorta.

After median sternotomy, the main PA was carefully dissected from the ascending aorta, and then banding tape was placed around the main PA using an umbilical tape. In PAB‐CPB group, the banding tape was gradually constricted over 2‐fold of RV systolic pressure at baseline data that was measured just before PAB. In Ctrl and CPB groups, the banding tape was just looped around the PA without constriction. A CPB with a roller peristaltic pump console (Sarns 8000) and a hollow fiber membrane oxygenator (CX‐RX05RW, Terumo) was established by central cannulation via the ascending aorta (12 Fr) and right atrium (20–24 Fr) after systemic heparinization (300 IU/kg). CPB was initiated, and the animals were perfused with CPB arterial pump flow rate of 100–120 ml/kg/min for 2 h at normothermia (36–37.5°C). The pigs were maintained a pH of 7.35–7.45, an arterial pco_2_ of 35–45 mmHg and an arterial po_2_ of >120 mmHg. After 2 h of CPB support, perfusion flow of CPB was decreased gradually, and then CPB was weaned. Blood glucose was measured using a Bayer Contour point‐of‐care glucometer (Bayer HealthCare) at regular intervals.

### 2.3 Infusion of carbon‐13 ( ^13^C)‐labeled substrates and tissue extraction

^13^C‐labeled substrates were used as tracers for metabolic analysis. [2‐^13^C]lactate and [2,4,6,8‐^13^C]octanoate, medium‐chain fatty acid (MCFA), were obtained from Sigma, and [U‐^13^C]mixed long‐chain fatty acids (LCFAs) were obtained from Cambridge Isotope Laboratories. ^13^C‐labeled substrates were infused into the right coronary artery for the final 60 min of the protocol. The intracoronary doses were adjusted to achieve 1.2 mM [2‐^13^C]lactate, 0.1 mM [2,4,6,8‐^13^C]octanoate and 0.1 mM [U‐^13^C]LCFAs concentrations based on the mean coronary artery flow per body weight calculated in our preliminary pig experiments (Kajimoto et al., 2013; Kajimoto, Ledee, et al., 2014; Kajimoto, O'Kelly Priddy, et al., 2014; Olson et al., 2008). Immediately upon completion of the ^13^C‐labeled infusion for 60 min, RV tissue in the region perfused by the right coronary artery was harvested and rapidly stored under liquid nitrogen for later examinations.

### 2.4 Nuclear magnetic resonance (NMR)

^13^C‐ and ^1^H‐NMR were performed on the RV extract tissue for measuring the fractional contribution (Fc) of each substrate to the acetyl‐CoA pool entering the citric acid cycle (CAC) and for measuring the concentration of myocardial energy metabolites respectively as previously described (Des Rosiers et al., 2004; Kajimoto et al., 2013, 2018, 2019).

^13^C‐NMR data were acquired on a Varian Direct Drive (VNMRS) 600 MHz spectrometer (Agilent Technologies) equipped with a Dell Precision T3500 Linux workstation running VNMRJ 4.0. The spectrometer system was outfitted with a Varian triple resonance salt‐tolerant cold probe with a cold carbon preamplifier. Fourier‐transformed spectra were fitted with commercial software (NUTS, Acorn NMR Inc.), and then the data which determined from specific carbon glutamate labeling were analyzed by the CAC analysis‐fitting algorithm tcaCALC (kindly provided by the Advanced Imaging Research Center at the University of Texas, Southwestern). Collected spectra by ^1^H‐NMR were analyzed using Chenomx software (version 8.3, Chenomx) with quantifications based on spectral intensities relative to 0.5 mM 2,2‐dimethyl‐2‐silapentane‐5‐sulfonate, which was added as a spike to each sample.

### 2.5 Measurement of systemic cytokine levels

Arterial blood samples were collected in ethylenediaminetetraacetic acid tubes at the end of protocol (approximately 4 h after CPB initiation) and centrifuged at 3000 rpm and 4°C for 10 min. The aliquot supernatant (plasma) was stored at −80°C for further analysis. Cytokine levels in plasma were analyzed using the Eve Technologies Discovery Assay Pig Cytokine Array (Eve Technologies): interleukin (IL)‐1β, 6, 8, 10, and tumor necrosis factor (TNF) α. Data were shown as the ratio of pro‐ and anti‐inflammatory cytokines.

### 2.6 Statistical analysis

Reported values are median (interquartile range) in the text, figures, and table. Statistical analysis was performed using PRISM 8 (Graph Pad Software Inc.). A paired *t*‐test was used for significant differences from the baseline value for each group in Table 1. Spearman test was used for evaluation of correlations between hemodynamic data and Fc of substrate. Two‐way ANOVA was used for evaluation of RV‐to‐LV systolic pressure ratio (RVSP/LVSP) of group and time and their interaction. Tukey's multiple comparisons test was used to assess mean post hoc differences at baseline and at end point between 3 groups. Other data were compared among 3 groups and analyzed with the non‐parametric Kruskal‐Wallis test with Dunn's multiple comparison test. The criterion for significance was *p* < 0.05 for all comparisons.

**TABLE 1:** Blood and tissue concentration

	Baseline (before CPB)			*p*	End point			*p*	Paired *t*‐test		
	Ctrl *n* = 5	CPB *n* = 5	PAB‐CPB *n* = 6		Ctrl *n* = 5	CPB *n* = 5	PAB‐CPB *n* = 6		Ctrl	CPB	PAB‐CPB
Blood											
Hemoglobin (g/dl)	9.5 (3.0)	10.5 (1.9)	10.5 (2.3)	0.69	9.9 (2.1)	9.1 (2.1)	8.6 (2.1)	0.19	0.56	0.01	0.01
Glucose (mg/dl)	86 (3)	74 (32)	83 (24)	0.87	103 (26)	113 (45)	122 (51)	0.26	0.30	0.01	0.02
RV tissue normalized by total creatine											
Glucose					0.13 (0.11)	0.15 (0.02)	0.15 (0.05)	0.25			
Glutamate					0.33 (0.19)	0.28 (0.06)	0.32 (0.06)	0.77			
Glutamine					0.50 (0.10)	0.44 (0.21)	0.39 (0.25)	0.37			
Alanine					0.16 (0.05)	0.16 (0.06)	0.17 (0.07)	0.88			
Lactate					0.13 (0.03)	0.18 (0.08)	0.12 (0.11)	0.53			
Carnitine					0.031 (0.014)	0.043 (0.007)	0.029 (0.018)	0.08			
Leucine					0.014 (0.006)	0.016 (0.005)	0.014 (0.003)	0.46			

*Note*: Results given as median (interquartile range). *p*, Probability value is by the Kruskal‐Wallis test among 3 groups.

Abbreviations: CPB, cardiopulmonary bypass; PAB, pulmonary artery banding.

## 3 RESULTS

The median (interquartile range) age and preoperative body weight were similar among 3 groups (Ctrl; 46 [15] days, 11.6 [3.1] kg, CPB; 37 [6], 12.3 [1.3] kg, PAB‐CPB; 38 [13] days, 12.6 [3.4] kg). There were no operative or technical complications in any pigs. The animals did not receive any blood transfusions or inotropic or vasoactive drugs. Blood hemoglobin and glucose levels were not statistically different among three groups at baseline (before starting CPB) and end point of the protocol (Table 1). Hemoglobin and blood glucose levels at end point of protocol are decreased and increased by the usage of CPB, respectively.

### 3.1 Cardiac parameters

Figure 1b,c showed parameters of cardiac function measured at an endpoint of study based on data before starting CPB. As expected, RV systolic pressure in PAB‐CPB group was significantly increased after weaning from CPB, and the end point value (54 [9] mmHg in PAB‐PAB) was also significantly higher than that in CPB group (30 [5] mmHg). RVSP/LVSP was markedly increased in PAB‐CPB group from 0.36 (0.06) at the baseline to 0.70 (0.06) at the end point (Figure 1d). RVSP/LVSP at the end point in PAB‐CPB group was significantly higher than RVSP/LVSP in Ctrl and CPB groups at the end point. High RVSP/LVSP in PAB‐CPB was due to high RVSP with normal LVSP in 4 cases and with low LVSP in 1 case. RPP, an indirect index of myocardial oxygen consumption, in RV was also higher in PAB‐CPB group than in Ctrl and CPB groups. The usage of CPB produced higher systemic inflammation (higher IL‐6/IL‐10), and PAB with CPB did not increase inflammatory response over CPB alone (Figure 1e).

### 3.2 Fc of ^13^C‐labeled substrates to CAC in RV

**FIGURE 2:**
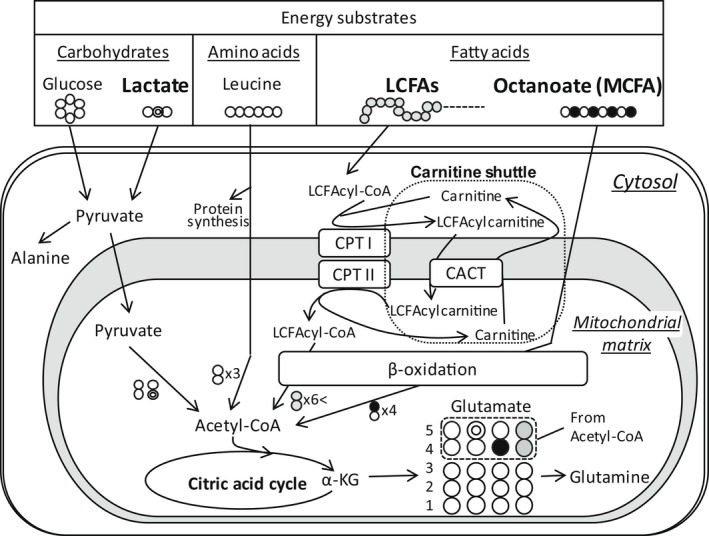
Labeling patterns of acetyl‐CoA and glutamate originating from metabolism of [2‐^13^C]lactate, [2,4,6,8‐^13^C]octanoate and [U‐^13^C]LCFAs. Full and double circles represent ^13^C and empty circle ^12^C. CACT, carnitine‐acylcarnitine translocase; CPT, carnitine palmitoyltransferase; LCFAs, long‐chain fatty acids; MCFA, medium‐chain fatty acid; α‐KG, α‐ketoglutarate.

**FIGURE 3:**
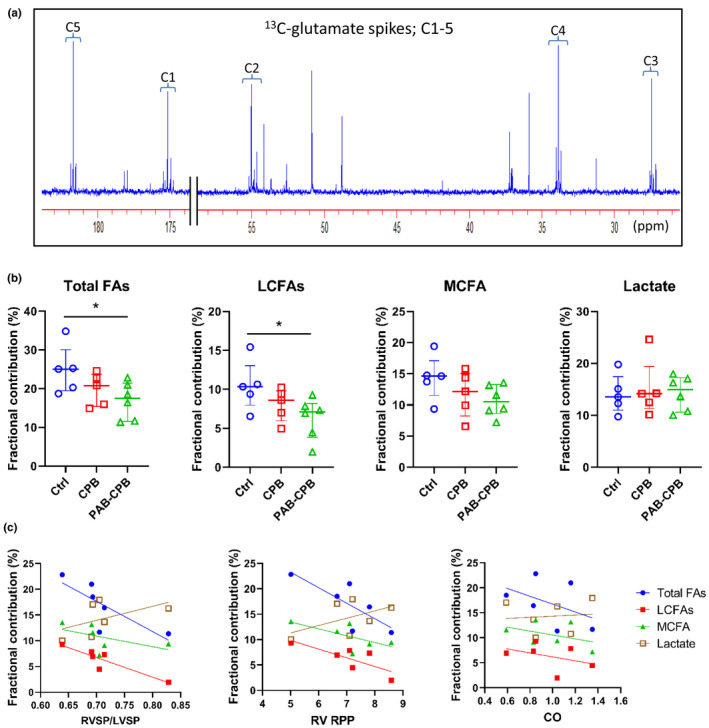
Fractional contribution from each ^13^C‐labeled substrate to acetyl‐CoA in RV by ^13^C‐NMR. (a) Typical ^13^C‐NMR full spectrum. Chemical shifts in parts per million (ppm) were as follows: C3, 27.5; C4, 33.8; C2, 55.0; C1, 175.0; C5 of glutamate, 181.7. (b) Fcs of total FA (LCFAs + MCFA) and LCFAs were statistically low in PAB‐CPB group compared with Ctrl group. Neither CPB nor PAB affected Fc of lactate. (c) Correlation of RVSP/LVSP and RV RPP to Fc of substrates in piglets received PAB and CPB (*n* = 6). These parameters were strongly correlated with Fc of total FA (LCFAs + MCFA). Values are median (interquartile range); *n* = 5–6 per group. *p < 0.05. CPB, cardiopulmonary bypass; Fc, fractional contribution; LCFAs, long‐chain fatty acids; MCFA, medium‐chain fatty acid; PAB, pulmonary artery banding; RPP, rate‐pressure product; RVSP/LVSP, RV‐to‐LV systolic pressure ratio; Total FAs, MCFA + LCFAs.

^13^C‐NMR data provide the absolute Fc for individual labeled substrates from glutamate labeling pattern with ^13^C (Figure 2). In this study, ^13^C‐NMR showed clear individual spectra for glutamates carbon 1–5 (Figure 3a). The unlabeled component consists of circulating substrates taken up by the heart, or endogenous substrates within the heart undergoing oxidation. Total Fc from ^13^C‐labeled substrates was around 35%. Fc for LCFAs in PAB‐CPB group was markedly lower than that in Ctrl group (Figure 3b). Thus, acute RVPO after the completion of CPB inhibited LCFA entering into the CAC, whereas Fc for MCFA and lactate to the CAC in RV was similar among the three groups. However, the hemodynamic response to RVPO after CPB was highly variable in PAB‐CPB group, we performed correlation studies to determine the relationship of RVSP/LVSP and RV RPP to substrate utilization. RVSP/LVSP and RV RPP following RVPO markedly correlated to Fc for total FA (LCFAs + MCFA) inversely, but not MCFAs or lactate oxidation (Figure 3c; Table 2). In addition, RVSP/LVSP tended to correlate to Fc for LCFAs inversely. Fc data from labeled substrates were analyzed from the ^13^C‐labeling pattern of glutamate. Therefore, RV tissue concentrations of glutamate and glutamine were measured by ^1^H‐NMR. These concentrations were not significantly different among the three groups (Table 1). In addition, lactate, alanine (pyruvate metabolites), and leucine (branched chain amino acid) in RV were similar among three groups. The level of carnitine, which is responsible for transporting LCFAs to the mitochondria, was higher in CPB group compared with other two groups (Table 1; Figure 2). However, this trend did not reach statistical significance (*p* = 0.08).

**TABLE 2:** Spearman correlation *r* and *p*‐values for associations between substrate oxidation and hemodynamic parameter in PAB‐CPB group

	RVSP/LVSP		RV RPP		CO	
	*r*	*p*	*r*	*p*	*r*	*p*
Total FAs	−0.9429	0.008	−0.8857	0.033	−0.2571	0.66
LCFAs	−0.8286	0.058	−0.7143	0.14	−0.2000	0.71
MCFA	−0.7714	0.10	−0.7143	0.14	−0.2571	0.66
Lactate	0.5429	0.30	0.3714	0.50	0.1429	0.80

Abbreviations: CO, cardiac output; CPB, cardiopulmonary bypass; LCFAs, long‐chain fatty acids; MCFA, medium‐chain fatty acid; PAB, pulmonary artery banding; RPP, rate‐pressure product; RVSP/LVSP, RV systolic pressure to LV systolic pressure ratio.

### 3.3 Energy components in RV

**FIGURE 4:**
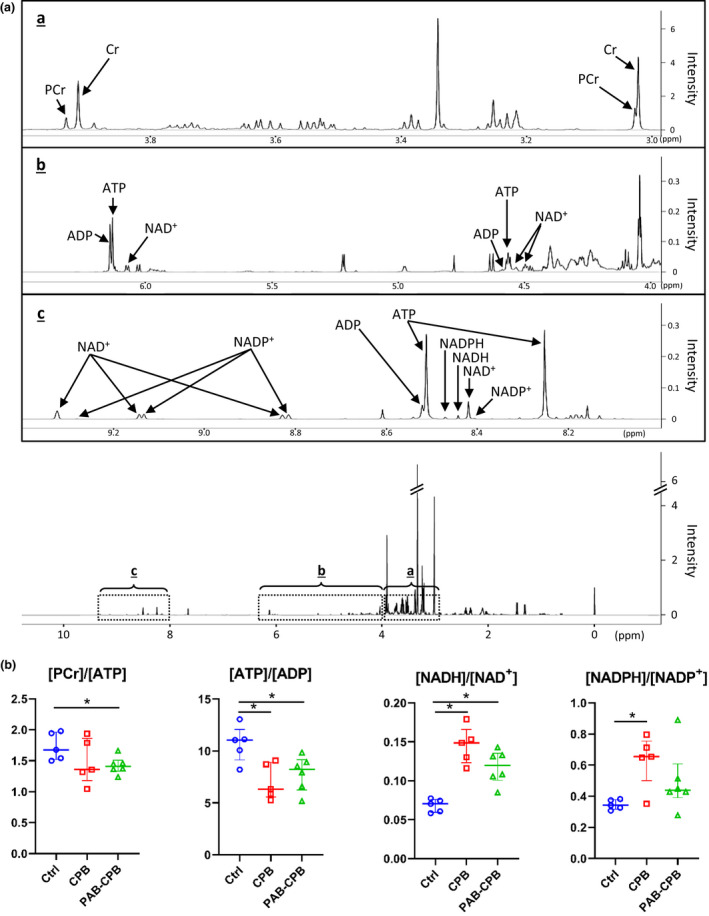
RV energy components by ^1^H‐NMR. (a) Typical ^1^H‐NMR spectrum for energy metabolites. a–c showed expansion spectrum at high frequency regions. (b) The RV [PCr]/[ATP] in CPB‐PAB group was significantly lower than that in Ctrl group. The RV [NADH]/[NAD^+^] in all 2 groups which used CPB was significantly higher than that in Ctrl group, whereas RV [NADPH]/[NADP^+^] was significantly higher in CPB group than Ctrl group. Values are median (interquartile range); *n* = 5–6 per group. *p < 0.05. ADP, adenosine diphosphate; ATP, adenosine triphosphate; CPB, cardiopulmonary bypass; NAD^+^, the oxidized form of nicotinamide adenine dinucleotide; NADH, the reduced form of nicotinamide adenine dinucleotide; NADPH, nicotinamide adenine dinucleotide phosphate; PCr, phosphocreatine.

We measured RV (Phosphocreatine [PCr])/(adenosine triphosphate [ATP]), (ATP)/(adenosine diphosphate [ADP]), reduced/oxidized ratio of nicotinamide adenine dinucleotide ([NADH]/[NAD^+^]) and adenine dinucleotide phosphate ([NADPH]/[NADP^+^]) demonstrating stability in the cellular energy state using ^1^H‐NMR (Figure 4). The RV [PCr]/[ATP], an index of cellular phosphorylation potential, and [ATP]/[ADP] were significantly low in PAB‐CPB group compared with Ctrl group. RV [ATP]/[ADP] in CPB group was also significantly higher than in Ctrl. The RV [NADH]/[NAD^+^], an index of redox status was higher in two groups which used CPB than Ctrl group. [NADPH]/[NADP^+^], the main modulator of the pentose phosphate pathway, was increased in CPB group compared with Ctrl; however, [NADPH]/[NADP^+^] in PAB‐CPB group was similar to Ctrl.

## 4 DISCUSSION

Prior studies have shown that the immature RV exhibits an imbalance between ATP production and ATP utilization during acute RVPO (Kajimoto et al., 2018). Under various loading and inotropic conditions, the RV demonstrates an inability to switch substrate preference compared to the LV (Kajimoto et al., 2017). This low metabolic flexibility during acute RVPO contributes to a decline in free energy of ATP hydrolysis, illustrated by decreasing [PCr]/[ATP]. In following, the energy deficit contributes to functional decline, and ultimate hemodynamic collapse as the [PCr]/[ATP] drops below the tolerable threshold.

Previously, we showed that mechanical circulatory support increases relative FA oxidation in the LV, while reciprocally decreasing citric acid flux through pyruvate dehydrogenase (Kajimoto et al., 2013). Shear‐stress associated with CPB inducing cytokine surge with modulation of insulin sensitivity and other hormonal responses are implicated for these metabolic changes (Floh et al., 2020; Tu et al., 2021). However, the unloaded RV does not illustrate similar metabolic flexibility (Kajimoto et al., 2017). In the current study, we observed in CPB group that mild decreases in relative FA flux, though not significant in our experiments, were adequate to induce imbalance in energy supply demand as illustrated by decrease in [ATP]/[ADP] and increase in [NADH]/[NAD^+^]. However, these metabolic shifts were not adequate to reduce [PCr]/[ATP], which serves as a more robust and accurate assessment of energy balance than [ATP]/[ADP].

CPB associated with inflammation was then superimposed on acute RVPO. Cytokines themselves can modulate cardiac function, and thereby the ventricular pressures, irrespective of FA oxidation (Prabhu, 2004). Although we noted the expected increase in cytokines between Ctrl and CPB groups, the ratios of pro‐ and anti‐inflammatory cytokines were not significantly different between CPB and PAB‐CPB groups. The loaded RV weaned from CPB exhibited modest decreases in free FA oxidation without concomitant reciprocal increase in lactate oxidation reflective of pyruvate dehydrogenase flux. This inability to increase flux through pyruvate dehydrogenase concurrent with diminishing FA oxidation contributed to further energy deficit and resulted in lower critical high energy phosphate ratios. Impairment in FA oxidation appears directly proportional to the degree of RVPO. In this protocol, the RV was able to fully recover baseline cardiac output even after PAB for a 3‐h period. However, continued depletion of high energy phosphate reserves would likely contribute to low cardiac output syndrome as commonly occurs between 6 and 18 h after congenital heart surgery in neonates and infants (Drennan et al., 2021; Wernovsky et al., 1995).

The RV inability to modify flux through pyruvate dehydrogenase is a consistent finding in experiments using various animal models (Bogh et al., 2020; Kajimoto et al., 2019). Previously, we have linked the reduction in pyruvate dehydrogenase flux to increased inhibiting phosphorylation of the pyruvate dehydrogenase. Following chronic PAB which produced RV hypertrophy in juvenile pigs, dichloroacetate stimulation of pyruvate dehydrogenase flux improved RV function (Kajimoto et al., 2019). These data in the hypertrophied RV suggest that oxidative capacity is not impaired. However, the signaling for the shifts in substrate flux, necessary for maintaining appropriate energy balance, is inadequate for increasing ATP production in the face of increasing demand.

It remains unclear why the pressure overloaded RV shows impaired FA oxidation and loss of activation of carbohydrate oxidation as typically noted with a Randle cycle type response. We need to target the regulators of FA and carbohydrate oxidations to define the mechanism of these processes. Studies have reported that peroxisome proliferator‐activated receptor α (PPAR‐α) levels decrease and activation of PPAR‐α restored FA oxidation in rodent pressure overload heart failure (Kaimoto et al., 2017) and in an ischemia–reperfusion model (Liepinsh et al., 2013). Recent data have also indicated an essential role for mitochondrial pyruvate carrier for sustaining carbohydrate oxidation in response to hemodynamic stress such as pressure overload (Fernandez‐Caggiano et al., 2020; Zhang et al., 2020). The current study supports our previous findings (Kajimoto et al., 2019) that metabolic manipulation such as stimulation of pyruvate dehydrogenase flux might benefit the immature RV exposed to acute stress such as occurs during congenital heart surgery. Alternatively, we have shown that increasing acetyl‐CoA flux to the CAC through provision of MCFAs circumvents disruption on energy metabolism caused by impairments in pyruvate dehydrogenase flux in the LV (Kajimoto et al., 2015). However, further research is required to determine response of the pressure overloaded RV exposed to CPB.

### 4.1 Study limitations

First, the current study used a relatively modest stressor, mechanical circulation, in addition to RVPO. We presume that addition of ischemia–reperfusion as occurs with congenital heart surgery procedures would further deplete energy reserves and eventually lead to marked dysfunction. We did not maintain experiments long enough to document this eventual dysfunction but have previously demonstrated this phenomenon in the non‐hypertrophied RV exposed to acute pressure loading. Secondly, in order to obtain substrate labeling required to achieve reasonable signal in the NMR experiment, we performed selective coronary perfusion. With the complexity of the model, we were unable to also perfuse the left coronary artery. Therefore, we do not have simultaneous metabolic data from the LV. However, we have shown in prior experiments that the LV under these conditions exhibits much greater metabolic flexibility than the RV. Lastly, sex differences, and especially sex hormones, may affect myocardial energy metabolism (Salerni et al., 2015). This study used only male pigs, although examination of sex as a biological factor influencing our results will be an area of future research.

## 5 CONCLUSIONS

The acutely loaded immature RV exhibits poor metabolic flexibility after weaning from CPB. FA oxidation decreases without reciprocal increase in flux through pyruvate dehydrogenase. This FA metabolic abnormality correlated to RV‐to‐LV systolic pressure ratio inversely. This leads to energy imbalance, which could eventually cause severe RV dysfunction consistent with low cardiac output syndrome noted in infants undergoing CPB procedures for congenital heart disease.

## AUTHOR CONTRIBUTIONS

Masaki Kajimoto, Muhammad Nuri, and Michael A. Portman designed the study. Masaki Kajimoto, Muhammad Nuri, Justin R. Sleasman, Kevin A. Charette, and Hidemi Kajimoto performed the experiments. Masaki Kajimoto and Hidemi Kajimoto analyzed the data and prepared the figures. Masaki Kajimoto and Michael A. Portman drafted the manuscript. All authors edited and revised the manuscript. All authors have read and approved the final version of the manuscript.

## FUNDING INFORMATION

Research reported in this publication was supported by the National Heart Lung and Blood Institute of the National Institutes of Health under award number R01HL60666 (M. A. P.). The content is solely the responsibility of the authors and does not necessarily represent the official views of the National Institutes of Health.

## CONFLICT OF INTEREST

No conflicts of interest, financial or otherwise, are declared by the authors.

## ETHICS STATEMENT

We used 1 month‐old male mixed breed Yorkshire piglets (Premier BioSource, Ramona, CA). All experimental procedures were reviewed and approved by Seattle Children's Research Institute Institutional Animal Care and Use Committee in compliance with guidelines from the Association for Assessment and Accreditation of Laboratory Animal Care. The care and use of animals also met the standards set by the National Institutes of Health for experimental animals.

## References

[phy215421-bib-0001] Bogh, N. , Hansen, E. S. S. , Omann, C. , Lindhardt, J. , Nielsen, P. M. , Stephenson, R. S. , Laustsen, C. , Hjortdal, V. E. , & Agger, P. (2020). Increasing carbohydrate oxidation improves contractile reserves and prevents hypertrophy in porcine right heart failure. Scientific Reports, 10, 8158. 32424129 10.1038/s41598-020-65098-7 PMC7235019

[phy215421-bib-0002] Bolger, A. P. , Sharma, R. , Li, W. , Leenarts, M. , Kalra, P. R. , Kemp, M. , Coats, A. J. , Anker, S. D. , & Gatzoulis, M. A. (2002). Neurohormonal activation and the chronic heart failure syndrome in adults with congenital heart disease. Circulation, 106, 92–99. 12093776 10.1161/01.cir.0000020009.30736.3f

[phy215421-bib-0003] Celermajer, D. S. , Cullen, S. , & Deanfield, J. E. (1993). Impairment of endothelium‐dependent pulmonary artery relaxation in children with congenital heart disease and abnormal pulmonary hemodynamics. Circulation, 87, 440–446. 8425291 10.1161/01.cir.87.2.440

[phy215421-bib-0004] Des Rosiers, C. , Lloyd, S. , Comte, B. , & Chatham, J. C. (2004). A critical perspective of the use of (13)C‐isotopomer analysis by GCMS and NMR as applied to cardiac metabolism. Metabolic Engineering, 6, 44–58. 14734255 10.1016/j.ymben.2003.10.004

[phy215421-bib-0005] Drennan, S. E. , Burge, K. Y. , Szyld, E. G. , Eckert, J. V. , Mir, A. M. , Gormley, A. K. , Schwartz, R. M. , Daves, S. M. , Thompson, J. L. , Burkhart, H. M. , & Chaaban, H. (2021). Clinical and laboratory predictors for the development of low cardiac output syndrome in infants undergoing cardiopulmonary bypass: A pilot study. Journal of Clinical Medicine, 10, 712. 33670210 10.3390/jcm10040712 PMC7916966

[phy215421-bib-0006] Fernandez‐Caggiano, M. , Kamynina, A. , Francois, A. A. , Prysyazhna, O. , Eykyn, T. R. , Krasemann, S. , Crespo‐Leiro, M. G. , Vieites, M. G. , Bianchi, K. , Morales, V. , Domenech, N. , & Eaton, P. (2020). Mitochondrial pyruvate carrier abundance mediates pathological cardiac hypertrophy. Nature Metabolism, 2, 1223–1231. 10.1038/s42255-020-00276-5 PMC7610404 33106688

[phy215421-bib-0007] Floh, A. A. , McCrindle, B. W. , Manlhiot, C. , Nakada, M. , La Rotta, G. , Van Arsdell, G. , & Schwartz, S. M. (2020). Feeding may modulate the relationship between systemic inflammation, insulin resistance, and poor outcome following cardiopulmonary bypass for pediatric cardiac surgery. JPEN. Journal of Parenteral and Enteral Nutrition, 44, 308–317. 30887547 10.1002/jpen.1529

[phy215421-bib-0008] Graham, T. P., Jr. , Bernard, Y. D. , Mellen, B. G. , Celermajer, D. , Baumgartner, H. , Cetta, F. , Connolly, H. M. , Davidson, W. R. , Dellborg, M. , Foster, E. , Gersony, W. M. , Gessner, I. H. , Hurwitz, R. A. , Kaemmerer, H. , Kugler, J. D. , Murphy, D. J. , Noonan, J. A. , Morris, C. , Perloff, J. K. , … Sutherland, J. L. (2000). Long‐term outcome in congenitally corrected transposition of the great arteries: A multi‐institutional study. Journal of the American College of Cardiology, 36, 255–261. 10898443 10.1016/s0735-1097(00)00682-3

[phy215421-bib-0009] Haller, C. , Friedberg, M. K. , & Laflamme, M. A. (2020). The role of regenerative therapy in the treatment of right ventricular failure: A literature review. Stem Cell Research & Therapy, 11, 502. 33239066 10.1186/s13287-020-02022-w PMC7687832

[phy215421-bib-0010] Kaimoto, S. , Hoshino, A. , Ariyoshi, M. , Okawa, Y. , Tateishi, S. , Ono, K. , Uchihashi, M. , Fukai, K. , Iwai‐Kanai, E. , & Matoba, S. (2017). Activation of PPAR‐alpha in the early stage of heart failure maintained myocardial function and energetics in pressure‐overload heart failure. American Journal of Physiology. Heart and Circulatory Physiology, 312, H305–H313. 28011586 10.1152/ajpheart.00553.2016

[phy215421-bib-0011] Kajimoto, M. , Ledee, D. R. , Isern, N. G. , & Portman, M. A. (2017). Right ventricular metabolism during venoarterial extracorporeal membrane oxygenation in immature swine heart in vivo. American Journal of Physiology. Heart and Circulatory Physiology, 312, H721–H727. 28159812 10.1152/ajpheart.00835.2016 PMC5407156

[phy215421-bib-0012] Kajimoto, M. , Ledee, D. R. , Olson, A. K. , Isern, N. G. , Des Rosiers, C. , & Portman, M. A. (2015). Differential effects of octanoate and heptanoate on myocardial metabolism during extracorporeal membrane oxygenation in an infant swine model. American Journal of Physiology. Heart and Circulatory Physiology, 309, H1157–H1165. 26232235 10.1152/ajpheart.00298.2015 PMC4631540

[phy215421-bib-0013] Kajimoto, M. , Ledee, D. R. , Olson, A. K. , Isern, N. G. , Robillard‐Frayne, I. , Des Rosiers, C. , & Portman, M. A. (2016). Selective cerebral perfusion prevents abnormalities in glutamate cycling and neuronal apoptosis in a model of infant deep hypothermic circulatory arrest and reperfusion. Journal of Cerebral Blood Flow and Metabolism, 36, 1992–2004. 27604310 10.1177/0271678X16666846 PMC5094314

[phy215421-bib-0014] Kajimoto, M. , Ledee, D. R. , Xu, C. , Kajimoto, H. , Isern, N. G. , & Portman, M. A. (2014). Triiodothyronine activates lactate oxidation without impairing fatty acid oxidation and improves weaning from extracorporeal membrane oxygenation. Circulation Journal, 78, 2867–2875. 25421230 PMC5570456

[phy215421-bib-0015] Kajimoto, M. , Nuri, M. , Isern, N. G. , Robillard‐Frayne, I. , Des Rosiers, C. , & Portman, M. A. (2018). Metabolic response of the immature right ventricle to acute pressure overloading. Journal of the American Heart Association, 7, e008570. 29848498 10.1161/JAHA.118.008570 PMC6015375

[phy215421-bib-0016] Kajimoto, M. , Nuri, M. , Isern, N. G. , Robillard‐Frayne, I. , Des Rosiers, C. , & Portman, M. A. (2019). Metabolic response to stress by the immature right ventricle exposed to chronic pressure overload. Journal of the American Heart Association, 8, e013169. 31450994 10.1161/JAHA.119.013169 PMC6755848

[phy215421-bib-0017] Kajimoto, M. , O'Kelly Priddy, C. M. , Ledee, D. R. , Xu, C. , Isern, N. , Olson, A. K. , & Portman, M. A. (2013). Extracorporeal membrane oxygenation promotes long chain fatty acid oxidation in the immature swine heart in vivo. Journal of Molecular and Cellular Cardiology, 62, 144–152. 23727393 10.1016/j.yjmcc.2013.05.014 PMC3739709

[phy215421-bib-0018] Kajimoto, M. , O'Kelly Priddy, C. M. , Ledee, D. R. , Xu, C. , Isern, N. , Olson, A. K. , & Portman, M. A. (2014). Effects of continuous triiodothyronine infusion on the tricarboxylic acid cycle in the normal immature swine heart under extracorporeal membrane oxygenation in vivo. American Journal of Physiology. Heart and Circulatory Physiology, 306, H1164–H1170. 24531815 10.1152/ajpheart.00964.2013 PMC3989754

[phy215421-bib-0019] Koop, A. M. C. , Hagdorn, Q. A. J. , Bossers, G. P. L. , van Leusden, T. , Gerding, A. , van Weeghel, M. , Vaz, F. M. , Koonen, D. P. Y. , Sillje, H. H. W. , Berger, R. M. F. , & Bartelds, B. (2019). Right ventricular pressure overload alters cardiac lipid composition. International Journal of Cardiology, 287, 96–105. 31003793 10.1016/j.ijcard.2019.04.004

[phy215421-bib-0020] Levy, D. , Laghlam, D. , Estagnasie, P. , Brusset, A. , Squara, P. , & Nguyen, L. S. (2021). Post‐operative right ventricular failure after cardiac surgery: A cohort study. Frontiers in Cardiovascular Medicine, 8, 667328. 34195233 10.3389/fcvm.2021.667328 PMC8236513

[phy215421-bib-0021] Liepinsh, E. , Skapare, E. , Kuka, J. , Makrecka, M. , Cirule, H. , Vavers, E. , Sevostjanovs, E. , Grinberga, S. , Pugovics, O. , & Dambrova, M. (2013). Activated peroxisomal fatty acid metabolism improves cardiac recovery in ischemia‐reperfusion. Naunyn‐Schmiedeberg's Archives of Pharmacology, 386, 541–550. 10.1007/s00210-013-0849-0 23525500

[phy215421-bib-0022] Norozi, K. , Wessel, A. , Alpers, V. , Arnhold, J. O. , Geyer, S. , Zoege, M. , & Buchhorn, R. (2006). Incidence and risk distribution of heart failure in adolescents and adults with congenital heart disease after cardiac surgery. The American Journal of Cardiology, 97, 1238–1243. 16616033 10.1016/j.amjcard.2005.10.065

[phy215421-bib-0023] Olson, A. K. , Hyyti, O. M. , Cohen, G. A. , Ning, X. H. , Sadilek, M. , Isern, N. , & Portman, M. A. (2008). Superior cardiac function via anaplerotic pyruvate in the immature swine heart after cardiopulmonary bypass and reperfusion. American Journal of Physiology. Heart and Circulatory Physiology, 295, H2315–H2320. 18849332 10.1152/ajpheart.00739.2008 PMC2614545

[phy215421-bib-0024] Piao, L. , Fang, Y. H. , Cadete, V. J. , Wietholt, C. , Urboniene, D. , Toth, P. T. , Marsboom, G. , Zhang, H. J. , Haber, I. , Rehman, J. , Lopaschuk, G. D. , & Archer, S. L. (2010). The inhibition of pyruvate dehydrogenase kinase improves impaired cardiac function and electrical remodeling in two models of right ventricular hypertrophy: Resuscitating the hibernating right ventricle. Journal of Molecular Medicine (Berlin, Germany), 88, 47–60. 10.1007/s00109-009-0524-6 PMC3155251 19949938

[phy215421-bib-0025] Prabhu, S. D. (2004). Cytokine‐induced modulation of cardiac function. Circulation Research, 95, 1140–1153. 15591236 10.1161/01.RES.0000150734.79804.92

[phy215421-bib-0026] Prisco, S. Z. , Hartweck, L. M. , Rose, L. , Lima, P. D. A. , Thenappan, T. , Archer, S. L. , & Prins, K. W. (2022). Inflammatory glycoprotein 130 signaling links changes in microtubules and junctophilin‐2 to altered mitochondrial metabolism and right ventricular contractility. Circulation. Heart Failure, 15, e008574. 34923829 10.1161/CIRCHEARTFAILURE.121.008574 PMC8766918

[phy215421-bib-0027] Reddy, S. , & Bernstein, D. (2015). Molecular mechanisms of right ventricular failure. Circulation, 132, 1734–1742. 26527692 10.1161/CIRCULATIONAHA.114.012975 PMC4635965

[phy215421-bib-0028] Salerni, S. , Di Francescomarino, S. , Cadeddu, C. , Acquistapace, F. , Maffei, S. , & Gallina, S. (2015). The different role of sex hormones on female cardiovascular physiology and function: Not only oestrogens. European Journal of Clinical Investigation, 45, 634–645. 25845675 10.1111/eci.12447

[phy215421-bib-0029] Schulze‐Neick, I. , Penny, D. J. , Rigby, M. L. , Morgan, C. , Kelleher, A. , Collins, P. , Li, J. , Bush, A. , Shinebourne, E. A. , & Redington, A. N. (1999). L‐arginine and substance P reverse the pulmonary endothelial dysfunction caused by congenital heart surgery. Circulation, 100, 749–755. 10449698 10.1161/01.cir.100.7.749

[phy215421-bib-0030] Sheikh, A. M. , Barrett, C. , Villamizar, N. , Alzate, O. , Valente, A. M. , Herlong, J. R. , Craig, D. , Lodge, A. , Lawson, J. , Milano, C. , & Jaggers, J. (2009). Right ventricular hypertrophy with early dysfunction: A proteomics study in a neonatal model. The Journal of Thoracic and Cardiovascular Surgery, 137, 1146–1153. 19379982 10.1016/j.jtcvs.2008.09.013

[phy215421-bib-0031] Sun, X. Q. , Zhang, R. , Zhang, H. D. , Yuan, P. , Wang, X. J. , Zhao, Q. H. , Wang, L. , Jiang, R. , Jan Bogaard, H. , & Jing, Z. C. (2016). Reversal of right ventricular remodeling by dichloroacetate is related to inhibition of mitochondria‐dependent apoptosis. Hypertension Research, 39, 302–311. 26763846 10.1038/hr.2015.153

[phy215421-bib-0032] Tu, L. N. , Hsieh, L. , Kajimoto, M. , Charette, K. , Kibiryeva, N. , Forero, A. , Hampson, S. , Marshall, J. A. , O'Brien, J. , Scatena, M. , Portman, M. A. , Savan, R. , Benner, C. , Aliseda, A. , Nuri, M. , Bittel, D. , Pastuszko, P. , & Nigam, V. (2021). Shear stress associated with cardiopulmonary bypass induces expression of inflammatory cytokines and necroptosis in monocytes. JCI Insight, 6, e141341. 10.1172/jci.insight.141341 PMC7821587 33232305

[phy215421-bib-0033] Wernovsky, G. , Wypij, D. , Jonas, R. A. , Mayer, J. E., Jr. , Hanley, F. L. , Hickey, P. R. , Walsh, A. Z. , Chang, A. C. , Castaneda, A. R. , Newburger, J. W. , & Wessel, D. L. (1995). Postoperative course and hemodynamic profile after the arterial switch operation in neonates and infants. A comparison of low‐flow cardiopulmonary bypass and circulatory arrest. Circulation, 92, 2226–2235. 7554206 10.1161/01.cir.92.8.2226

[phy215421-bib-0034] Zhang, Y. , Taufalele, P. V. , Cochran, J. D. , Robillard‐Frayne, I. , Marx, J. M. , Soto, J. , Rauckhorst, A. J. , Tayyari, F. , Pewa, A. D. , Gray, L. R. , Teesch, L. M. , Puchalska, P. , Funari, T. R. , McGlauflin, R. , Zimmerman, K. , Kutschke, W. J. , Cassier, T. , Hitchcock, S. , Lin, K. , … Abel, E. D. (2020). Mitochondrial pyruvate carriers are required for myocardial stress adaptation. Nature Metabolism, 2, 1248–1264. 10.1038/s42255-020-00288-1 PMC8015649 33106689

